# Exhausted Grape Seed Residues as a Valuable Source of Antioxidant Molecules for the Formulation of Biocompatible Cosmetic Scrubs

**DOI:** 10.3390/molecules28135049

**Published:** 2023-06-28

**Authors:** Yara Salem, Hiba N. Rajha, Suhair Sunoqrot, Alaa M. Hammad, Ines Castangia, Maria Manconi, Maria Letizia Manca, Dana Al Lababidi, Joe A. Touma, Richard G. Maroun, Nicolas Louka

**Affiliations:** 1 Centre d’Analyses et de Recherche, Unité de Recherche Technologies et Valorisation Agro-Alimentaire, Faculté des Sciences, Université Saint-Joseph de Beyrouth, Riad El Solh, P.O. Box 17-5208, Beirut 1104 2020, Lebanon; yara.salem1@net.usj.edu.lb (Y.S.); dana.lababidi@net.usj.edu.lb (D.A.L.); nicolas.louka@usj.edu.lb (N.L.); 2 Département de Génie Chimique et Pétrochimique, Faculté d’Ingénierie, Ecole Supérieure d’Ingénieurs de Beyrouth (ESIB), Université Saint-Joseph de Beyrouth, CST Mkalles Mar Roukos, Riad El Solh, Beirut 1107 2050, Lebanon; hiba.rajha@usj.edu.lb; 3 Department of Pharmacy, Faculty of Pharmacy, Al-Zaytoonah University of Jordan, P.O. Box 130, Amman 11733, Jordan; suhair.sunoqrot@zuj.edu.jo (S.S.); alaa.hammad@zuj.edu.jo (A.M.H.); 4 Department of Life and Environmental Sciences, University of Cagliari, University Campus, S.P. Monserrato-Sestu Km 0.700, 09042 Monserrato, CA, Italy; ines.castangia@unica.it (I.C.); manconi@unica.it (M.M.); 5 Château Saint Thomas, Bekaa Valley, Kab Elias, Lebanon; joe@closstthomas.com

**Keywords:** grape seeds, polyphenols, antioxidants, sustainability, natural scrub, by-products, cosmeceuticals, circular economy

## Abstract

Grape seed of Obeidi, a white Lebanese autochthonous variety, was previously tested in different studies as a valuable source of bioactive molecules such as polyphenols, oils, and proteins by means of extraction procedures for the development of cosmetic and therapeutic products. However, an un-valorized, exhausted grape seed residue remains as “secondary waste” after the extraction processes. In this study, the exhausted seeds have been further exploited to produce cosmetic scrubs capable of releasing antioxidant molecules during the exfoliation process, in accordance with the principles of the circular economy and going toward a zero-waste process. The deep characterization of the exhausted seeds confirmed the presence of antioxidant phenolic molecules including gallic acid, catechins and protocatechuic acid (0.13, 0.126, and 0.089 mg/g of dry matter DM), and a high phenolic content (11.85 mg gallic acid equivalents (GAE)/g of dry matter (DM)). Moreover, these residues were shown to possess a sandy texture (Hausner ratio (HR): 1.154, Carr index (CI): 0.133, and angle of repose: 31.62 (°) degrees), similar to commercial natural exfoliants. In this respect, exhausted Obeidi grape seed residues were incorporated at increasing concentrations (0.5, 1, 1.5, and 2% *w*/*w*) in a cosmetic scrub, and stored for 5 weeks at 4, 25, and 50 °C for stability testing. All tested scrub formulations exhibited good spreadability with a spread diameter of 3.6–4.7 cm and excellent physical stability, as no phase separation or color change were observed after four cycles of heat shock at 4 and 50 °C. Finally, an in vivo skin irritation test showed that the scrub enriched with 1.5% of exhausted Obeidi grape seed residues was the most promising formulation, as it possessed a high amount of phenolic molecules (0.042 ± 0.001 mg GAE/mL of scrub) and good stability and could be safely applied to the skin with no irritation phenomena. Overall results underlined that exhausted grape seed residues can be transformed into promising systems for both physical and chemical exfoliation, thus confirming the importance of the effective exploitation of agro-industrial by-products for the development of high value cosmeceutics towards a more sustainable and zero-waste approach.

## 1. Introduction

Grape, according to the Food and Agriculture Organization (FAO), is one of the largest fruit crops in the world, and is associated with the production of a considerable amount of solid waste [1]. Indeed, the production of grape pomaces in Mediterranean countries is about 1200 tons per year, which are mostly used as soil improvers or to produce animal feed. Unfortunately, a large part of these pomaces is still dumped in open spaces due to connected economic issues, causing environmental pollution [2]. These ecological concerns are mainly related to the low pH of pomaces, due to the presence of phenolic compounds [3], leading to resistance to biological degradation and soil and water pollution [4,5]. Moreover, leachates of tannins and other chemicals may cause oxygen depletion in the soil, thus affecting their flora’s growth and health [6]. To address these issues, the scientific community is constantly looking for alternative and innovative strategies capable of valorizing this biomass by turning it into high-added-value food supplements, nutraceuticals, or cosmeceutical products [7,8], due to their high content of active molecules such as polyphenols, lipids (oils), and proteins [9,10,11].

Among the different bio-residues obtained after wine production, grape seeds are the richest in phytochemicals such as polyphenols, including anthocyanins, flavanols, stilbenes, phenolic acids [12,13], and flavonoids, which possess a wide range of biological and beneficial properties [14,15]. These active molecules have been tested for the treatment of a wide variety of human diseases; they have been demonstrated to be capable of preventing skin disorders [16], intestinal diseases [17], and even neurodegenerative diseases, mostly connected with oxidative stress and inflammatory processes [18,19]. Moreover, The CosIng (Cosmetic Ingredients) database lists nine different types of *Vitis-Vinifera*-derived raw materials that can be used in cosmetic products. For example, grape seed extract is suggested as a humectant, coloring agent, and hair and skin conditioner [20]. Grape-based cosmetics have many anti-aging properties and do not irritate the skin [21]. Nowadays, many natural cosmetics companies are using raw materials from food sector waste, and they are putting more effort into developing cosmetics with a low negative impact on the environment. It was found that mango seed kernels, grinded olive stones, and *Moringa peregrina* seeds are used as exfoliating powders in many cosmetic applications, but no study or application was reported about using grape seed residues in a cosmetic scrub [22].

In light of all the promising results, grape seed residues might be used as functional ingredients in cosmetic and cosmeceutical products, as they ensure the homeostasis of the skin and the prevention of skin diseases, especially those induced by external factors such as sun exposure, chill, and dust [23] that might intensify skin aging and pigmentation, and also favor roughness and wrinkles [24]. To reduce, slow down, and prevent skin aging, the use of a scrub enriched with active and beneficial molecules capable of eliminating all the impurities and deeply cleaning the skin seems to be an ideal strategy. Exfoliation or deep peeling can boost collagen production, which is the key factor in healthy, bright skin [25], enhance skin firmness and elasticity, and reduce the visibility of fine lines and wrinkles [26]. Therefore, exfoliation, from both chemical and physical points of view, is the process responsible for cleaning and removing the excess of corneocytes (differentiated Keratinocytes) accumulated in the stratum corneum along with all the impurities, which can lead to more radiant and smoother skin [27]. Alpha (α)-hydroxy acids (AHAs), including lactic, glycolic, and malic acids, or β-hydroxy acids, including salicylic acid, are the most commonly used chemical peels that can effectively remove the excess of keratinocytes and stimulate the growth of new epidermal cells [25,28]. However, physical exfoliation, by using scrubs with abrasive solid particles, can induce immediate and effective desquamation of skin cells [27] and can be used as an alternative or in combination with chemical exfoliators for a better synergistic effect. Regardless of the scrub type used (facial or body scrub), it must be non-toxic, non-irritant, and non-sticky [29], but it must also be mildly abrasive and possess small, gritty particles capable of removing dead skin cells [30]. Moreover, the scrub must ensure the elimination of dead skin cells and enhance skin hydration. To reach all these beneficial properties, the use of natural plant-based scrubs may represent an ideal strategy, as plants or parts of them are rich in active molecules (antioxidants, vitamins, essential oils, etc.); in particular, grape extracts have previously proven their ability to rejuvenate and revitalize skin cells [31].

This study aims first at finding a natural plant-based by-product scrub as an alternative to those commonly used that contain essential nutritional foods (sugar, salt) [32] or synthetic polymeric particles (polyethylene (PE), polypropylene (PP)), which may in turn cause toxicity for the environment [33]. Secondly, it finds a sustainable solution for the wine industry, helping in reducing their waste; thus, the cosmetic industries are seeking to employ sustainable and biodegradable skin- and eco-friendly raw materials in cosmetics, such as seeds from fruits [34]. The third objective is to create a natural scrub rich in natural antioxidant molecules that also promotes both physical and chemical exfoliation. For that, after the incorporation of the natural grape seed residues at different concentrations into a basic commercial formula as a scrub, all formulas were tested for the following *in vitro* stability tests: heat-shock cycles, pH, viscosity, and spread ability. They were also tested for their polyphenol release by means of total phenolic content and antioxidant activity. Moreover, an in vivo skin irritation test was performed to evaluate the real tolerability of the final product once applied to the skin. The scrub formulations produced might therefore act as a catalyst for growth in the cosmetic industry.

## 2. Results

### 2.1. Chemical Composition and Antioxidant Activity of the Obeidi Grape Seed Residues

**Figure 1:**
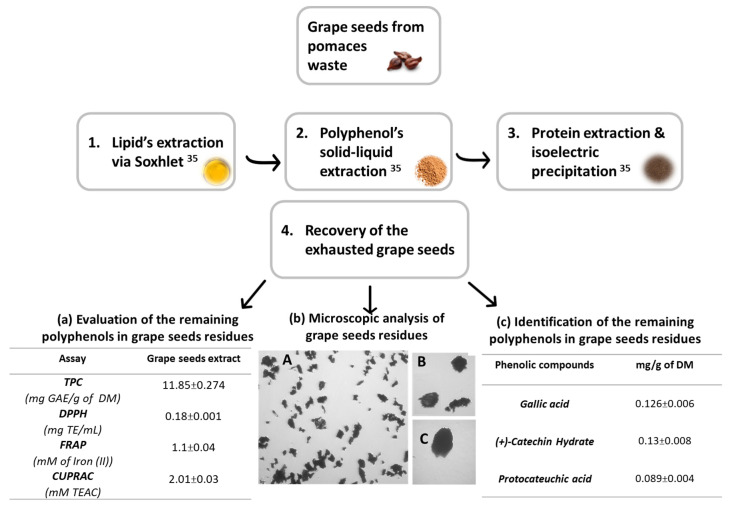
Schematic representation of the multistep fractionation of grape seeds from pomaces waste, (**a**) total phenolic content (TPC) still contained in grape seed residues and the antioxidant activity (evaluated by means of DPPH, FRAP, and CUPRAC colorimetric assays), (**b**) microscopic images of the Obeidi grape seed residues at 4× (A), 10× (B), and 20× (C) magnification using an optical microscope, and (**c**) the identification and quantification of the remaining phenolic compounds (HPLC-DAD technique).

In a previous study, a multistep fractionation of Obeidi grape seeds from pomace waste (first waste) was done as represented in Figure 1. After the extraction of lipids (12–13%), polyphenols (6.5–12.2%), and proteins (3.9–4.1%), a significant amount of the remaining grape seed residues (60–65%) was recovered and tested in terms of physicochemical properties to evaluate their possible incorporation into cosmetic scrubs targeting a zero-waste approach [35]. Obeidi grape seed residues (second waste) were subjected to a further solid-liquid extraction to evaluate the total phenolic content and the antioxidant activity of the remaining phenolics in these residues. The total phenolic content (TPC) was around 11.85 ± 0.274 mg gallic acid equivalent (GAE)/g of dry matter (DM), with antioxidant activity of 0.18 ± 0.001 mg/mL of Trolox equivalents (TE) using the Diphenyl-2-picrylhydrazyl (DPPH) assay, 1.1 ± 0.04 mM of Iron (II) equivalents using the ferric reducing antioxidant power (FRAP) assay, and 2.01 ± 0.03 mM of Trolox equivalents using the cupric ion reducing antioxidant capacity (CUPRAC) assay (Figure 1a). Moreover, the remaining phenolic compounds in the Obeidi grape seed residues were identified and quantified using the high-performance liquid chromatography (HPLC) technique (Figure 1c). Among all, gallic acid, catechin hydrate, and protocatechuic acid, which are well-known phytochemicals with strong antioxidant, antimicrobial, anti-inflammatory, and anticancer activities [36], were detected in high amounts (0.126 ± 0.006, 0.13 ± 0.008, and 0.089 ± 0.004 mg/g DM, respectively).

### 2.2. Physical Characterization of the Obeidi Seed Residues

After the detection of the main active molecules and the evaluation of both phenolic content and antioxidant activity, the physical properties of the Obeidi seed residues were assessed to prove their suitability for use in cosmetic scrubs. Being the main component of the scrub, both the sphericity and shape of the grape seed residues were evaluated using an optical microscope at 4×, 10×, and 20× magnification (Figure 1b(A–C), respectively). All images proved that these residues had no sharp edges and were mostly spherical. The flowability and texture of the powder were detected by calculating the Carr’s index (*CI*) (Equation (5)), Hausner ratio (*HR*) (Equation (4)), and angle of repose (Equation (7)) [37]. Their properties were then compared to those of commercial natural exfoliant powders (raspberries and *Argania spinosa*), sugar, and sand. According to the results shown in Table 1, Obeidi grape seed residues had high fluidity (Carr’s index of 0.133) and a sandy consistency (Hausner ratio of 1.154), similar to that of commercial exfoliants (Carr’s Index of 0.161 and Hausner ratio of 1.192). The powder’s consistency was very similar to the one of berries, sugar, and sand used as references. As for the angle of repose, the powder flowability was acceptable for all powders, as the angle width ranged between 30 and 50 (°) degrees.

### 2.3. Spread Ability

After making sure that the powder was convenient for use in scrubs, the spread ability test of different formulas was carried out at different concentrations of grape seed residue (0, 0.5, 1, 1.5, and 2%). The latter refers to the ability of a formulation to be topically spread across the skin [38]. Wider contact between the scrub and the skin, due to its good spreading capacity, promotes the absorption of the contained bioactives into the skin. Table 2 presents the spread ability values of grape seed scrubs (0, 0.5, 1, 1.5, and 2%) at 3 different temperatures: 4 °C (fridge), 25 °C (room temperature), and 50 °C (oven). The diffusion diameter of the scrubs enriched with Obeidi grape seed residues (0, 0.5, 1, 1.5 and 2%) was influenced by the number of seeds added and varied between 3.6 ± 0.1 cm and 4.7 ± 0.1 cm.

### 2.4. Stability Tests

#### 2.4.1. Heat Shock Test

**Figure 2:**
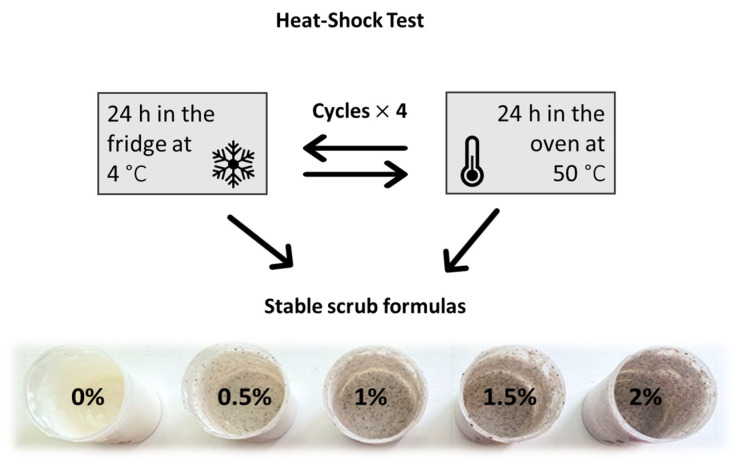
Schematic representation of the heat-shock test performed on scrubs enriched with different amounts of Obeidi seed residues (0, 0.5, 1, 1.5, and 2%).

After ensuring both physical and chemical characteristics of the Obeidi grape seed residues are incorporated in the scrubs, it is required to ensure the stability of the final formulated products at different temperatures by conducting different tests (heat-shock cycles, pH, viscosity, and shelf life). The stability of the scrubs enriched with Obeidi grape seed residues at different concentrations (0, 0.5, 1, 1.5, and 2%) was evaluated via the heating and cooling cycles. This method was performed to follow the changes in terms of physicochemical characteristics of the final formulation, such as phase separation, sedimentation of the solid components (grape seed particles), and changes in color, as a function of the temperature variations. Four cycles of cooling (4 °C in the fridge) and heating (50 °C in the oven) were performed (Figure 2). After this heat-shock test, no phase separation, coalescence, sedimentation, or color change was observed, irrespective of the concentration tested. Given that, exhausted Obeidi grape seed residues have proven to be rheologically stable when added to scrub formulas and, as a result, represent a significant advance for the skincare industry.

#### 2.4.2. pH & Viscosity Assays

**Figure 3:**
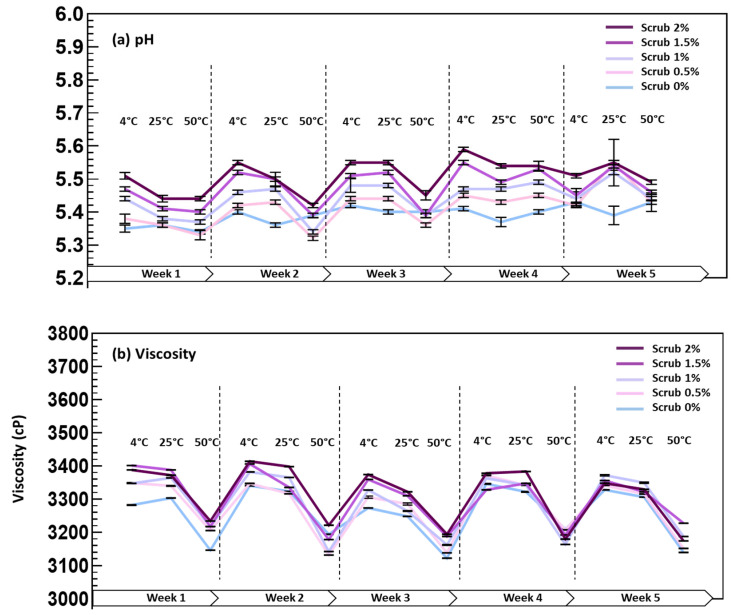
Measurements of pH (**a**) and viscosity (**b**) of the scrub batches enriched with different quantities of Obeidi seed residues (0, 0.5, 1, 1.5, and 2%) stored at 4 °C (fridge), 25 °C (room temperature), and 50 °C (oven) for 5 weeks. Mean values ± standard deviations were reported; *n* = 3.

The stability of the scrubs was also tested in terms of pH variation as a function of the temperature tested (4, 25, and 50 °C) during 5 weeks of storage (Figure 3). The pH of the scrubs enriched with Obeidi residues ranged from 5.3 to 5.5, with no significant differences between the formulas (*p* > 0.05) using the least significant difference (LSD). Additionally, the pH of the scrubs was stable over the tested period, with no significant variation across all temperatures at the same concentration.

The viscosity of the scrubs was monitored as a function of the temperature (4, 25, and 50 °C) during 5 weeks of storage (Figure 3). It was not only measured to evaluate the scrubbing effectiveness but also to assess the resistance of the scrubs enriched with Obeidi grape seed residues against the possible deformations that can occur at different temperatures. The viscosities of the enriched scrubs ranged between 3259 and 3314 cP (centipoise), with no significant difference between the ones evaluated at 4 and 25 °C (*p* > 0.05), for the same concentration. However, As expected, at 50 °C, the viscosity was slightly lower, but it remained stable during all 5 weeks of storage.

#### 2.4.3. Polyphenol Content and Antioxidant Activity

**Figure 4:**
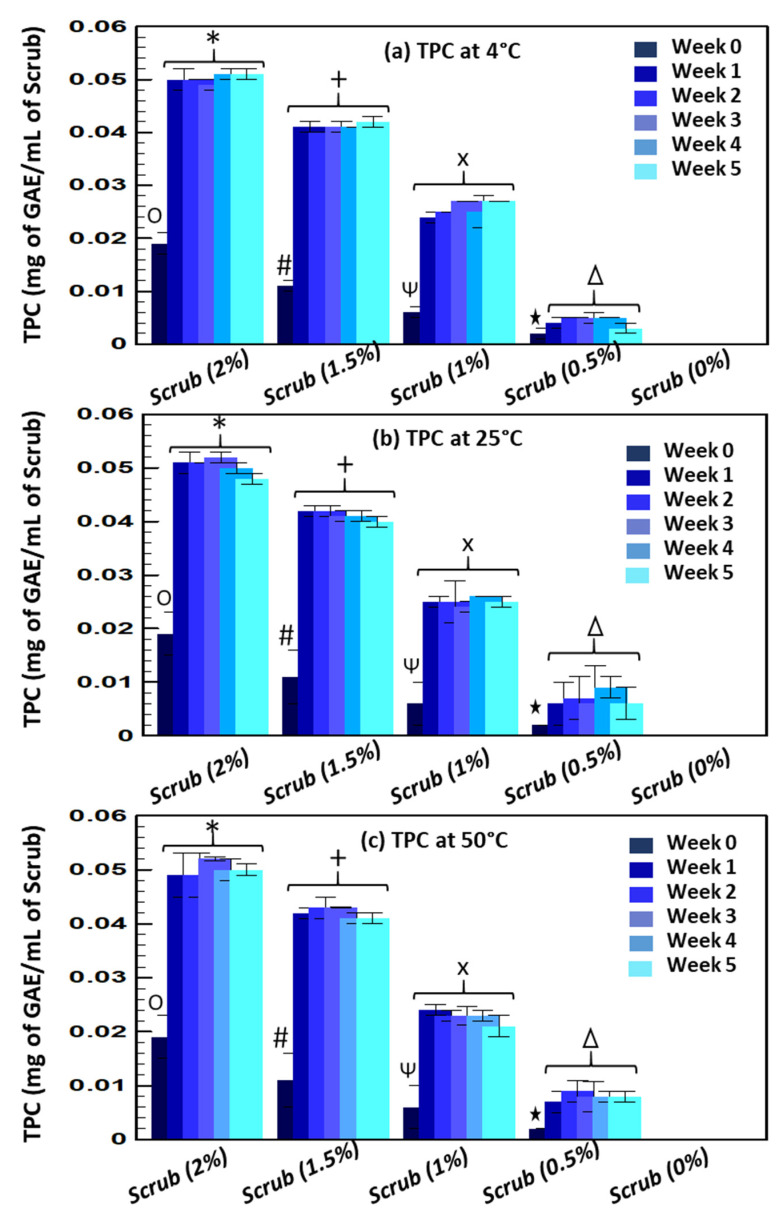
Total phenolic content (TPC) measured at 4 °C (**a**), 25 °C (**b**), and 50 °C (**c**) of the scrubs enriched with different quantities of Obeidi grape seeds residues (0, 0.5, 1, 1.5, and 2%) up to 5 weeks. Mean values ± standard deviations were reported. Same symbols indicate not statistically different values (*p* > 0.05).

All stability tests confirmed that the rheological properties of the scrubs were not affected by the addition of the Obeidi grape seed residues. Furthermore, to evaluate the possible beneficial properties of the enriched scrubs, the total phenolic content along with the antioxidant activity of the Obeidi grape seed residues were evaluated in the final product at different temperatures (4, 25, and 50 °C) for 5 weeks (Figure 4). After the preparation (week 0), all scrubs exhibited a low total phenolic content, which increased to a constant value the following weeks until week 5. The initial total phenolic content was 0.019 ± 0.004 mg GAE/mL for the scrub incorporating 2% Obeidi residues, 0.011 ± 0.05 mg GAE/mL for the 1.5% scrub, 0.006 ± 0.005 mg GAE/mL for the 1% scrub, followed by 0.002 ± 0.001 mg GAE/mL for the scrub with 0.5% Obeidi residues, while no phenolic compounds were detected in the basic formula (without Obeidi seed residues). Surprisingly, the total phenolic content increased significantly (*p* < 0.05) after 1 week, regardless of the temperature tested. Indeed, at 4 °C, the total phenolic content of the scrub enriched with exhausted Obeidi grape seed residues was ~3 times, ~4 times, ~3.8 times and ~2 times higher when 2%, 1.5%, 1%, and 0.5% of the residues were added, respectively, and remained stable over the 5-week period of testing (*p* > 0.05). Similarly, at 25 °C, the total phenolic content of the scrub enriched with Obeidi grape seed residues was ~2.5-fold, ~3.6-fold, ~4-fold and ~3-fold higher when 2%, 1.5%, 1%, and 0.5% of the residues were added. However, no statistically significant differences were found between weeks 1 and 5 (*p* > 0.05), as the phenolic content remained constant. At 50 °C, the behavior was similar, as the total phenolic content increased ~2.6-fold, ~3.7-fold, ~3.5-fold and ~4-fold for 2%, 1.5%, 1%, and 0.5% scrubs. However, no statistically significant differences were found between weeks 1 and 5 (*p* > 0.05), as the phenolic content remained constant. Regardless of the testing temperature (4, 25, or 50 °C), the scrub containing the highest amount of Obeidi grape seed residues (2%) had a higher total phenolic content during the 5-week period.

**Figure 5:**
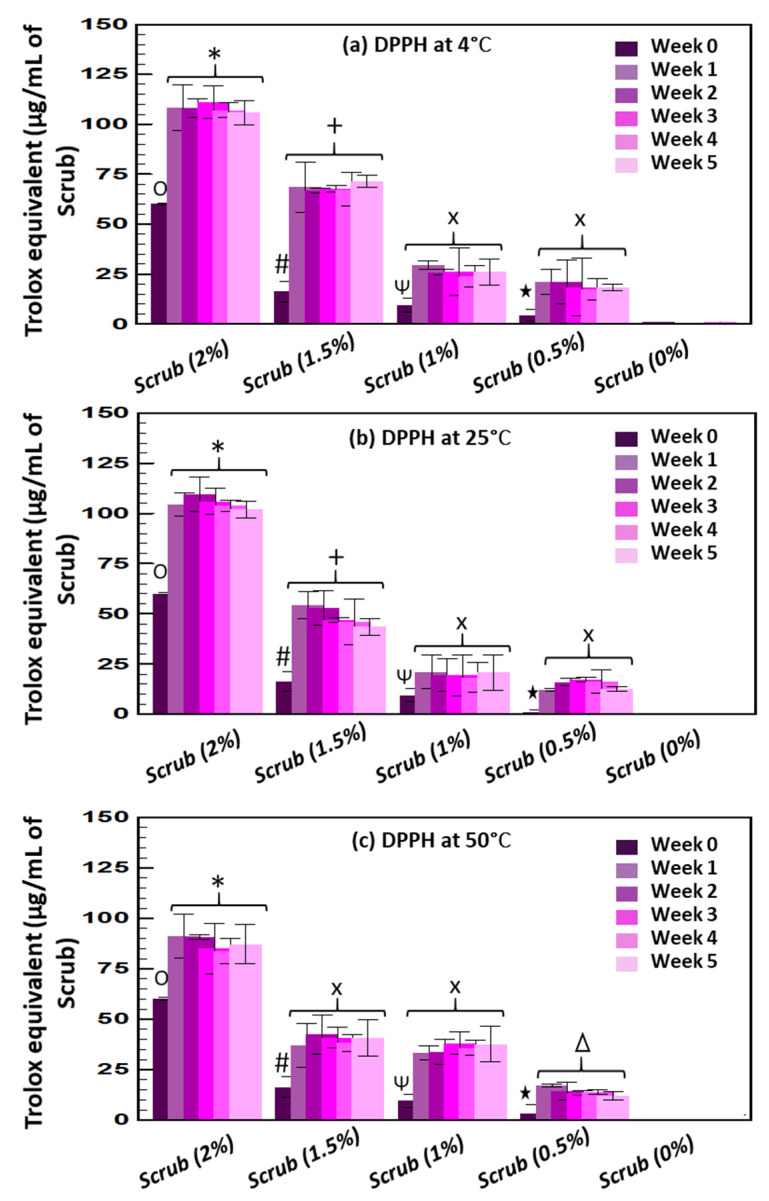
Antioxidant activity (DPPH assay) measured at 4 °C (**a**)**,** 25 °C (**b**) and 50 °C (**c**) of the scrubs enriched with different amount (0, 0.5, 1, 1.5, and 2%) of Obeidi grape seeds residues 5 weeks of storage. Mean values ± standard deviations were reported. Same symbols indicate not statistically different values (*p* > 0.05).

Following the evaluation of the scrubs’ total phenolic content, the antioxidant activity was evaluated as well using the DPPH colorimetric assay at different temperatures (4, 25, and 50 °C) and for 5 weeks of storage (Figure 5). Directly after their preparation, all tested enriched scrubs had minimal antioxidant activity at week 0, which significantly increased on day 7 and remained constant up to week 5. Indeed, the initial antioxidant activity was 60.10 ± 0.56, 16.32 ± 5.07, 9.55 ± 3.38, and 3.78 ± 2.5 µg/mL of Trolox equivalents for the scrub incorporating 2, 1.5, 1, and 0.5% of the Obeidi grape seed residues, respectively, whereas the basic formula (0% Obeidi seed residues) did not show any significant value during the whole storage period (5 weeks). After one week, the antioxidant activity of all scrub formulations was significantly increased at all temperatures tested. At 4 °C, after one week the antioxidant activity was ~1.7 times, ~4.4 times, ~2.7 times and ~4.7 times higher for 2, 1.5, 1, and 0.5% scrubs, respectively. No significant differences were observed across weeks, and the antioxidant activity of the scrubs remained constant (*p* > 0.05). Similarly, at day 7 and 25 °C, the antioxidant activity was ~1.7 times, ~2.7 times, ~2.17 times and ~1.9 times higher for 2, 1.5, 1, and 0.5% scrubs, respectively. At 50 °C, after 7 days, the antioxidant activity was ~1 times, ~2.5 times, ~3.9 times, and ~4 times higher for 2, 1.5, 1, and 0.5% scrubs, respectively. For all scrubs, both total phenolic content and antioxidant activity were significantly different (*p* < 0.05) between days 0 and 7, irrespective of the temperature tested, while any variation was further detected from day 7 up to the 5th week.

### 2.5. In Vivo Skin Irritation Test

**Figure 6:**
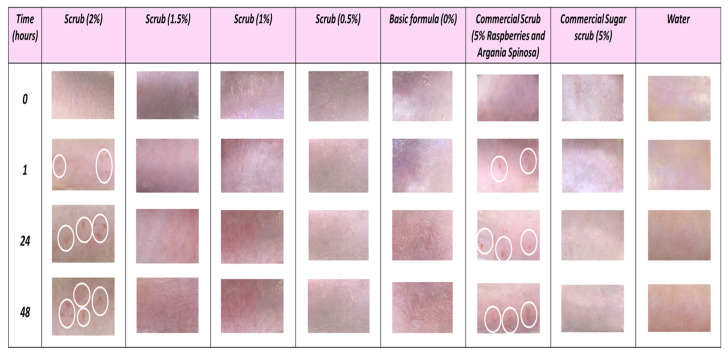
In vivo skin irritation test of the scrub enriched with different quantities (0, 0.5, 1, 1.5, and 2%) of exhausted Obeidi grape seed residues, compared to commercial natural and sugar-based scrubs (positive controls) and water (negative control) for 0, 1, 24, and 48 h.

To assess the safety of the formulated scrubs, an in vivo study was carried out on rats. Rats were divided into eight groups; five of them received the scrubs enriched with Obeidi grape seed residues at different concentrations (0.5, 1, 1.5, and 2%), with 0% being the basic commercial formula. One group was treated with a commercial natural scrub, another group was treated with a commercial sugar scrub as a positive control, and the last group was treated with water as a negative control. During the 48-hour (h) period, both water and the basic formulation (0% scrub) did not show any signs of redness, irritation, or induce any allergic reaction. Moreover, the commercial sugar scrub and the formulated scrubs containing the Obeidi grape seed residues of 0.5%, 1%, and 1.5% also showed no allergic reaction, inflammation, or swelling within the 48 h of the experiment. On the other hand, the scrub formulation incorporating 2% of the Obeidi grape seed residues and the commercial natural scrub showed some redness after 1 h where the soreness, irritation, and itching became more pronounced after each application and especially at 24 h and 48 h (Figure 6, white circles).

## 3. Discussion

**Figure 7:**
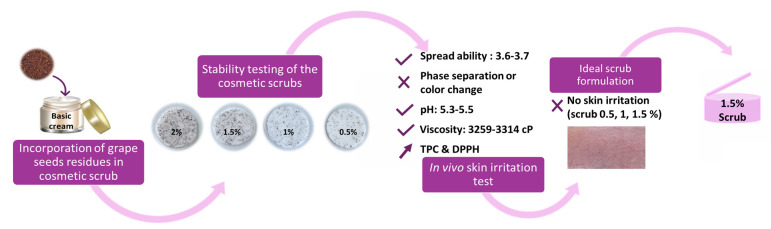
Schematic representation of Obeidi grape seed residue enriched- scrub formulation process.

Besides being a source of vitamins and fiber, grape seeds are rich in active molecules such as polyphenols, especially proanthocyanins, which can be used as functional ingredients to address various health problems by enhancing the body’s natural biological processes. Since grape seeds are by-products of wineries, they can be easily found and exploited as a valuable source of bioactive agents [16]. Antioxidant compounds are essential components widely used in cosmetic products such as body scrubs, since they provide protection against reactive oxygen species (ROS) that have deleterious effects on human skin [39]. Therefore, this study focuses on the use of exhausted Obeidi seeds as secondary residues for the development of enriched and effective scrubs, leading on the one hand to the complete exploitation of agri-food residues and on the other to the formulation of high-added-value products perfectly in line with zero waste and circular economy principles (Figure 7).

After the extraction of lipids, polyphenols, and proteins from grape seeds isolated from pomaces, the obtained residue was recovered and analyzed in terms of phytochemical composition and physicochemical properties to assess its possible incorporation into cosmetic scrubs. The TPC results show that some phenolic compounds were still present in the Obeidi seeds, which were also responsible for the antioxidant activity detected in this residue. The DPPH results indicate that the antioxidant mechanism of the grape seed extracts is carried out via hydrogen donor transfer, which can block the oxidation cycle by converting free radicals to stable forms [40]. Moreover, the CUPRAC and FRAP tests show that the antioxidants in the extracts can also donate electrons to reduce the oxidized forms of the lipid peroxidation process. The CUPRAC test is based on the antioxidants reducing Cu (II) to Cu (I), whereas the FRAP test is based on the antioxidants reducing Fe (II) to Fe (I) [40].

Gallic acid, catechin hydrate, and protocatechuic acid were the most abundant bioactives still contained in the grape seed residues, which are mainly responsible for the antioxidant activity, as previously reported by others [41]. Indeed, in previous studies, gallic acid was proven to be effective in reducing the expression of proteins linked to melanogenesis, including microphthalmia-associated transcription factor (MITF) and tyrosinase-related protein-1 (TRP1), and both tyrosinase activity and melanin production in a dose- and time-dependent manner, allowing it to be used as an adjuvant in various therapeutic formulations to reduce skin hyperpigmentation other than being a useful component in skin lightening and whitening products [42]. Even catechin, found at a concentration of 0.13 ± 0.008 mg/g of DM, has been shown to be a key component for the prevention of skin damage thanks to its capability of scavenging free radicals and reducing extracellular matrix degradation, mainly caused by pollution and ultraviolet (UV) radiation. Moreover, previous studies have demonstrated the ability of catechins to activate collagen synthesis and prevent the release of matrix metalloproteinase enzymes [43]. Similarly, protocatechuic acid (PCA), found at a concentration of 0.089 ± 0.004 mg/g of DM, also proved to have high antioxidant activity. It has also proven to be capable of inducing the synthesis of type I collagen in human dermal fibroblasts and inhibiting the secretion of matrix metalloproteinase MMP-1, which is involved in the breakdown of the collagen extracellular matrix. Previous studies have confirmed the potential of protocatechuic acid in the treatment of skin disorders both in vivo and in vitro [44]. All these results proved that the phenolic compounds of Obeidi grape seeds can act as an effective chemical exfoliant, antioxidant, and chelating agent. Moreover, according to Chandra et al., grape residues rich in antioxidants can be responsible for the revitalization of the skin, can also protect it from some types of skin cancer caused by ultraviolet radiation, and can reduce dark spots and wrinkles as well, both caused by uncontrolled free radicals’ activities [31].

Furthermore, grape seed residues are ideal to be incorporated into scrubs as the soft borders can avoid irritation or scratches while simultaneously ensuring an effective physical peel. Additionally, due to their physical properties, grape seed residues can replace some synthetic and plastic materials often used in scrubs, such as polyethylene and/or polypropylene beads, and can be a good alternative to commonly used exfoliants such as entire fruits (berries and pomegranate) or other essential food nutrients that are not likely to be used in cosmetics (sugar, salt, etc.) [32,33]. Grape seed’s granular texture helps physically exfoliate the skin, sloughing off dead skin cells and revealing a fresher, brighter skin complexion.

Further analyses showed that the spread ability of the formulated scrubs was similar to that reported by Desi E. et al. for coffee grounds in cosmetic formulations, according to which a spread’s diameter between 3 and 5 cm seemed to be ideal for cosmetics [45]. In all enriched-scrubs irrespective of the concentrations tested, the spread ability increased as a function of the temperatures, with the highest diameter observed at 50 °C, and decreased as a function of the quantity of Obeidi grape seed residues incorporated into the scrub, with the lowest diameter for the scrub enriched with the highest amount of residues (2%). Thus, the high amount of grape seed residue makes the scrub stiffer, enhancing its ability to deep clean and exfoliate the skin.

After the heat-shock test was performed to evaluate the stability of the scrub under different conditions, no phase separation (i.e., coalescence or sedimentation) or color change were observed for all tested concentrations. This is in agreement with previous studies performed by Talpekar et al. that confirmed the absence of any phase separation or color difference of the scrubs containing jojoba meal (1, 1.5, and 2%) when stored at different temperatures (4 and 45 °C) [46].

Furthermore, the pH is considered one of the key factors responsible for erythema and edema at the skin level [47]. The pH of the enriched scrubs was stable, ranged between 4 and 7, and met the specifications for a topical formulation. Similarly, Hilda et al. observed a pH ranging from 4.6 to 6.8 for the scrub formulation enriched with Arabica green coffee particles [48]. Additionally, a cosmetic product’s pH change may indicate instability, direct or indirect contamination during formulation, or potential chemical interactions between raw components [49].

During the entire storage period, all scrubs’ viscosities were found to be in accordance with the criteria placed by SNI 16-4399-1996 and ranged between 2000 and 50,000 centipoise (cP) [50]. For scrubs, the greater the increase in the viscosity of the formulation, which is the case with the grape seed scrubs, the more intense the scrubbing effect is.

Regardless of the temperature tested (4, 25, or 50 °C), the scrub containing the higher number of Obeidi grape seed residues (2%) showed the highest total phenolic content across the 5 weeks of testing. Based on these results, the Obeidi grape seed residues are still rich in active molecules, which were released especially during the first week after the preparation, knowing that the main ingredients of the scrub formulas are water and glycerin, which in previous studies have been shown to be efficient extraction solvents for polyphenols [51]. In light of this, the scrub enriched with Obeidi grape seed residues may be of great interest in cosmetic products containing phenolic compounds such as hydroxybenzoic acids (gallic acid), catechins, and protocatechuic acid, known for their beneficial effects on skin antiaging [52], lightening, hydration, and smoothness [53]. Furthermore, according to Kligman et al., a long-term phenol peel has a very long-lasting effect and can effectively reduce wrinkles, dark spots, freckles, and actinic keratose [54].

According to the Arrhenius equation for the shelf life of a product using the accelerated stability method, a product that is stable at 50 °C for one month is expected to have an eight-month shelf life at room temperature, which is the case for our formulated grape seed scrubs [55]. Over 5 weeks of testing, the scrub containing 2% Obeidi grape seed residues displayed the best antioxidant activity at 4, 25, and 50 °C. According to these findings and those of the TPC assay, polyphenols were released over the course of the 5 weeks when the scrubs were stored at 3 different temperatures, which explains the difference in peak levels between week 0 and the other 5 weeks. These results confirmed that all scrubs containing different concentrations of Obeidi grape seed residues possess a significant number of polyphenols released during the first weeks of storage. The DPPH assay confirmed the antioxidant activity of polyphenols contained in the scrub, enabling their use as potent anti-aging and exfoliating products [56,57].

Skin irritation after topical application is primarily defined as a localized, non-immunologically mediated inflammatory condition with no apparent visual or microscopic changes. Depending on the skin type, erythema, edema, vesiculation, burning, soreness, or itching might also appear [58,59]. The commercial sugar scrub and the formulated scrubs containing the exhausted Obeidi grape seed residues (0.5, 1, and 1.5%) showed no allergic reaction, inflammation, or swelling within the 48 h of the experiment, confirming their safety and high biocompatibility with the skin. On the other hand, the scrub formulation incorporating 2% of the Obeidi grape seed residues showed some redness after 1 h, and the soreness, irritation, and itching became more pronounced after each application, especially at 24 h and 48 h. These results were similar to those obtained with the commercial scrub containing more than 2% of a mixture of raspberries seeds and *Argania* shells in different shapes and sizes, confirming that skin irritation risk increases as the mass of the ground seeds in the formula also increases, while skin irritation risk decreases the lower the mass and the higher the dispersion of the abrasives are in the scrub formula. According to overall results, the formulated scrubs containing 1.5% of the Obeidi grape seed particles might be considered the ideal formulation incorporating, a satisfying amount of the residues with considerable antioxidant activity and stability along with pH and viscosity properties that can be spread and rubbed into the skin safely with no signs of irritation.

## 4. Materials and Methods

### 4.1. Plant Material

Obeidi grape pomace waste, kindly supplied by Château Saint Thomas (Bekaa Valley, Lebanon), was collected after winemaking as “first waste”. In a previous study [35], seeds were separated from skins, and subjected to subsequent extractions for the recovery of several bioactive molecules (lipids, polyphenols, and proteins), and the remaining residues, called “second waste”, were washed, filtered, and dehydrated in an airflow oven at 50 °C for 48 h, then stored in the dark at room temperature to be later on incorporated in cosmetic scrubs.

### 4.2. Chemicals

All experiments have been performed using analytical-grade chemicals. Folin-Ciocalteu solution, gallic acid, sodium carbonate, 6-hydroxy-2,5,7,8-tetramethylchroman-2-carboxylic acid 114 (Trolox), and 2,2-diphenyl-1-picrylhydrazyl (DPPH) and all HPLC Standards of gallic acid (3,4,5-Trihydroxybenzoic acid), *p*-Hydroxybenzoic acid, (+)-catechin hydrate, protocatechuic, rutin hydrate, quercetin (3,3’,4’,5,7-Pentahydroxyflavone), chlorogenic acid, p-coumaric acid, caffeic acid (3,4-Dihydroxycinnamic acid), trans-cinnamic acid, ellagic acid, resveratrol, and sinapic acid (3,5-Dimethoxy-4-hydroxycinnamic acid) were obtained from Sigma-Aldrich (Darmstadt, Germany). All assay kits for FRAP, CUPRAC, and proanthocyanin were purchased from Bioquochem (Asturias, Spain). As for the solvents, aqua and O-Phosphoric Acid 85% extra pure were provided by Fisher Scientific (Janssen Pharmaceuticalaan, Belgium), and Acetonitrile was provided by LiChrosolv^®^ (Darmstadt, Germany). Polyoxyethylene-20 (Tween 20) was obtained from Biotech (Markham, ON, Canada). A basic cream formula and two commercial exfoliating scrubs were purchased from a local store (Beirut, Lebanon) and used as positive controls, one containing 5% sugar and the other containing 5% raspberry seed powder and *Argania spinosa* shell powder.

### 4.3. Grape Seed Residues Chemical Composition and Physical Aspect

#### 4.3.1. Dry Matter

Five grams of the Obeidi grape seed residues were weighed and then placed in a drying oven (Nabertherm^®^ GmbH, Haan, Germany) set at 105 °C overnight. The dry matter content of the Obeidi seeds was 88.6 ± 0.1%.

#### 4.3.2. Microscopic Analysis

A few particles of Obeidi grape seed residues were observed on a Zeiss Primovert digital microscope (New York, NY, USA) and photographed using an Axiocam ERc 5s (New York, NY, USA) to evaluate the size and shapes of the particles at 4, 10, and 20× magnification.

#### 4.3.3. Extraction of the Remaining Polyphenols in Grape Seed Residues

The exhausted Obeidi grape seed residues were extracted, to quantify the remaining phenolic compounds in the “second waste”, using a mixture of ethanol and water (80:20 *v*/*v*) at a solid-to-liquid ratio of 1:5 (*w*/*v*). Grape seed residues were extracted in a digital water bath (JSR JSWB-22T, Gong Ju-city, Korea) for 2 h at 60 °C under continuous shaking, and the extract was then filtered through glass wool. The residual extract was diluted and used for the following assays [35].

#### 4.3.4. Evaluation of the Total Phenolic Content (TPC) of the Residual Grape Seeds Extract

The total phenolic content was evaluated with the Folin-Ciocalteu technique [60,61]. Eight hundred microliters of sodium carbonate (${Na}_{2}{CO}_{3}$, 75 g/L) were added to 200 µL of the diluted extracts and 1000 µL of (10%) the Folin-Ciocalteu solution. The mixture was incubated for 10 min at 60 °C, followed by 10 min of cooling at 4 °C. A spectrophotometer (UV192 Vis, Biochrom Ltd., Cambridge, UK) set at 750 nm was then used to measure the absorbance and calculate the total phenolic content. The extractive medium was used as a blank, and a calibration curve was built using gallic acid as a standard. The TPC value was represented as mg GAE/g of DM.

#### 4.3.5. Identification and Quantification of Phenolic Compounds in the Residual Grape Seed Extract by High-Performance Liquid Chromatography with Diode-Array Detection (HPLC-DAD)

The identification followed by the quantification of individual phenolic compounds remaining in exhausted Obeidi grape seed residues was carried out using an HPLC autosampler (module 1260 Vialsampler), where separation was obtained with a Discovery^®^ HS C18 column (25 cm × 4.60 mm, 5 µm, Supelco, Merck KGaA, Darmstadt, Germany) fitted with a Dionex RS diode array detector set at 280, 360, and 520 nm. A mobile phase A of 0.22 M phosphoric acid and phase B of 100% acetonitrile were used, with an injection volume of 10 µL and an average flow rate of 0.8 mL/min. The gradient was obtained by decreasing solvent A from 100% to 80% in 20 min, to 70% in 35 min, and then to 0% in 45 min remaining constant and stable up to 50 min [62]. Mixed standard solutions were prepared in ethanol at different concentrations (0.25–0.015 mg/mL), and calibration curves were built correlating the area of the peaks vs. the concentration. All samples were filtered before the injection through PTFE filters (0.22 μm, Ø 25 mm) and set in the autosampler.

#### 4.3.6. Evaluation of the Antiradical Activity of the Residual Grape Seeds Extract Using the Diphenyl-2-Picrylhydrazyl (DPPH) Free Radical Scavenging Method

The ability of phenolic compounds to reduce DPPH (2,2-diphenyl-picrylhydrazyl) was used to measure the free radical scavenging activity of the extract [63]. Fifty microliters of the grape extract was added to 1450 μL of DPPH solution (0.06 mM in methanol). The absorbance was measured at 515 nm after 30 min of incubation at room temperature (25 °C) in the dark. Fifty microliters of methanol (instead of the sample) was added to 1450 μL of DPPH solution and used as a blank. A calibration curve was built using Trolox (Sigma-Aldrich, St-Quentin Fallavier, France) as a standard. The extract’s inhibitory percentage (%) was estimated using the formula below:

(1) $$Inhibition\;percentage\;(\%)=\frac{\left(absorbance\;of\;negative\;control\;-\;absorbance\;of\;sample\right)}{absorbance\;of\;negative\;control}\times 100$$

Results were represented as milligram of Trolox equivalent per milliliters (mg TE/mL).

#### 4.3.7. Evaluation of the Antioxidant Activity of the Residual Grape Seed Extract Using Ferric Reducing Antioxidant Power Assay (FRAP)

The FRAP antioxidant capacity kit (Bioquochem, Asturias, Spain) was used to assess the antioxidant activity that can reduce the ferric complex at an acidic pH in the presence of antioxidant compounds [51]. According to the manufacturer’s protocol, 220 μL of the ready-to-use FRAP working solution was combined with 10 μL of diluted extract, or standard. After 4 min of mixing under constant stirring, the absorbance was measured at 593 nm using a plate reader. A calibration curve was built using iron II as a standard. The amount of iron (II) equivalents (mM) was used to measure the antioxidant activity.

#### 4.3.8. Evaluation of the Antioxidant Activity of the Residual Grape Seed Extract by Using Cupric ion Reducing Antioxidant Capacity Assay (CUPRAC)

To assess the antioxidant capacity of the extract, the CUPRAC test kit (Bioquochem, Asturias, Spain) was also used [51]. According to the manufacturer’s protocol, 200 μL of the working solution was mixed with 40 μL of the diluted extract, or standard (Trolox), and incubated for 30 min at room temperature. After incubation, the absorbance was measured at 450 nm using a microplate reader. A calibration curve was built using Trolox as a standard. Results were presented as millimoles of Trolox equivalents (mM TEAC).

### 4.4. Physical Evaluation and Characterization of the Grape Seed Residues

Grape seed residues’ physical characteristics were evaluated and compared to (i) commercial natural mix of raspberries’ seed powder and *Argania spinosa* shell powder, (ii) sugar, and (iii) sand, all used as references by applying the following methods:

#### 4.4.1. Hausner Ratio (HR) and Carr Index (CI)

A defined volume of the Obeidi grape seed residues was placed in a cylinder, and the corresponding mass was taken. The apparent density $\left(\rho_{apparent}\right)$ (bulk density) was then calculated using Equation (2). The same cylinder containing the sample with the same mass was then tapped, and the new volume was taken. The tapped density $\left(\rho_{tapped}\right)$ was then calculated using Equation (3) [37,64]:

(2) $$\rho_{apparent}(g/mL)=\frac{mass(g)}{volume(mL)}$$

(3) $$\rho_{tapped}(g/mL)=\frac{mass(g)}{volume\;tapped(mL)}$$

The powder diagnostic was then evaluated by calculating the Hausner ratio (*HR*) using Equation (4) [37]. The powder was considered sandy if the *HR* ≤ 1.25, fine if the 1.25 ≤ *HR* ≤ 1.4, and cohesive if the *HR* ≥1.4:

(4) $$HR=\frac{\rho_{tapped}}{\rho_{apparent}}$$

The flowability of the powder was assessed by calculating Carr’s compressibility index (*CI*) using Equation (5) [37]. The powder’s flowability was considered excellent if the *CI* was within the range of 0.05–0.15, good within 0.15–0.18, fair within 0.18–0.22, poor within 0.22–0.35, and poor if the *CI* > 0.35 [64]:

(5) $$CI=\frac{{\rho_{tapped}-\rho }_{apparent}}{\rho_{tapped}}$$

#### 4.4.2. Angle of Repose and Flow Rate

One hundred grams of Obeidi grape seed residues were placed in a funnel where the diameter of the internal orifice (*d*) was 10 mm, and the height of the cone (*H*) was 40 mm. The powder was then left to run on a blank piece of paper, and the adequate time was recorded in seconds to assess the flow rate of the powder (Equation (6)). The radius obtained was then measured in millimeters, the average diameter (*D*) was calculated over 3 repetitions, and the angle was calculated in degrees (Equation (7)). The flowability of the powder was considered excellent if the angle in degrees (°) was <30°, acceptable if the angle (°) was between 30 and 50°, and bad if the angle (°) was >50° [37]:

(6) $$Flow\;rate(g.s^{-1})=\frac{mass(g)}{time(s)}$$

(7) $$Angle({}^{\circ})=(\frac{Arc\tan\frac{\left(2\times H\right)}{\left(D-d\right)}}{2\times \pi })\times 360$$

### 4.5. Incorporation of Grape Seed Residues in Cosmetic Creams

Grape seed residues were added in various amounts (0.5, 1, 1.5, and 2% *w*/*w*) to a commercial basic formula that mainly consists of water, glycerin, stabilizers, and preservatives with no bioactive compounds. The commercial basic formula was chosen, as it was suitable for all skin types and provided long-lasting moisturization of the skin.

### 4.6. Spread Ability Tests

The petri plate was filled with 0.5 g of the tested formula, and the petri plate’s lid was reversed and then placed on top. At 25 °C, a load of 150 g was applied for 1 min over the lid before the spread’s diameter was determined. The spread diameter should be between 3 and 5 cm [45].

### 4.7. Stability Tests

Three batches of scrubs were prepared, each of which contained different percentages of grape seed residues (0, 0.5, 1, 1.5, and 2%). All scrubs were stored for more than 5 weeks at 3 different temperatures (4, 25, and 50 °C) [51].

#### 4.7.1. Heat-Shock Tests

Each scrub was kept at 4 °C for 24 h before being immediately removed and placed at 50 °C for another 24 h. Four cycles of this technique were completed for all formulas to detect possible instabilities such as precipitation, phase separation, or sedimentation [65].

#### 4.7.2. Viscosity and pH Tests

After the heating and cooling cycles, the physicochemical properties of the scrubs were evaluated in terms of viscosity and pH. The viscosity of the scrubs was measured at room temperature (25 °C) using a Brookfield DV-II + pro viscometer (Brookfield Engineering Laboratories Inc., Middleboro, MA, USA) equipped with an S64 spindle set to 100 rpm. The pH was measured at room temperature (25 °C) as well, using a pH meter (WTW, Frankfurt, Germany). All measurements were taken over a period of 5 weeks.

#### 4.7.3. Polyphenol Release: Total Phenolic Content (TPC) and Antioxidant Activity of the Scrubs Enriched with the Obeidi Residues

A solution of Polyoxyethylene-20 (Tween 20) in water (1:5 *v*/*v*) was first prepared, and then 3 mL of this solution was added to 1 mL of each scrub formula [66]. Following the dilution, the mixture was maintained under constant stirring at 400 rpm and 50 °C until it was completely transparent. The residues of the scrub were left to settle at the bottom of the beaker. For both TPC and antioxidant activity analysis, the clear supernatant solutions were collected. All measurements were taken over a period of 5 weeks for the 3 batches (4, 25, and 50 °C).

### 4.8. In Vivo Skin Irritation Test

The in vivo skin irritation test was performed in accordance with the ethical guidelines of the Animal Welfare Committee of the Al-Zaytoonah University of Jordan and with the Helsinki guidelines for animal research, and it was previously approved by Al-Zaytoonah University of Jordan Institutional Review Board (IRB) (decision no. 1/2/2020-2021). Healthy female rats weighing 150–200 g were divided into 8 groups (*n* = 2). Five of the animal groups were used to test the scrubs enriched with 0, 0.5, 1, 1.5, and 2% of the Obeidi grape seed residues. The remaining 3 groups were used to test a commercial natural scrub (raspberries’ seed powder and *Argania spinosa* shell powder), a commercial sugar-based scrub, and water, which were all used as controls. Prior to the test, the rats had 15 cm^2^ (3 cm wide and 5 cm high) shaved on their backs using an electric razor. Before each application, the back of the rat was cleaned with distilled water, and then approximately 0.5 g of the scrub was gently rubbed (5 circular movements) and kept on the skin until the next application. The skin reaction was evaluated at 0, 1, 24, and 48 h [45].

### 4.9. Statistical Analysis

Each graphic included a representation of the means and standard deviation (SD). STATGRAPHICS^®^ Centurion XVI program was used to perform the variance analysis (ONE-WAY ANOVA) and the least significant difference test (LSD) (StatPoint Technologies, Inc., Warrenton, VA, USA). The means that share the same letter are not statistically different from one another (with a *p* > 0.05). Each test was repeated three times.

## 5. Conclusions

An effective and complete exploitation of grape pomaces has been successfully achieved using exhausted grape seeds to formulate a cosmetic scrub containing different concentrations (0.5, 1, 1.5, and 2%) of this residue. All results showed that the latter may be safely added to scrub formulations up to 1.5% to accomplish mechanical exfoliation with no skin irritation. The most promising scrub formula showed good stability during the 5 weeks of storage at 4, 25, and 50 °C, with an average pH and viscosity of 5.47 and 3304.03 cP, respectively. Moreover, the phenolic content detected in all the scrubs tested contributes to the chemical exfoliation of the skin and provides a supply of antioxidants that can promote healthier-looking skin with anti-aging properties. This study succeeded in developing a natural cosmetic product using the unvalued leftover grape seed residues as a valuable source of bioactives in line with a sustainable and innovative strategy toward a zero-waste process. Finally, to confirm the scrub’s effectiveness, more investigations about its microbiological stability are required.

## Figures and Tables

**Table 1:** Physical characterization of the exhausted Obeidi grape seed residues and comparison with sugar, sand, and a commercial natural exfoliant. Results are reported as mean ± SD, *n* = 3.

	Obeidi Grape Seed Residues	Commercial Natural Exfoliant (Raspberry Seed Powder and *Argania spinosa*)	Commercial Sugar Exfoliant	Sand (Reference)
	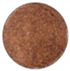	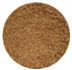	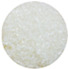	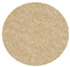
Particle size (mm)	2.00−4.75	2.00−4.75	2.00−4.75	2.00−4.75
*ρ* (apparent) (g/mL)	0.437 ± 0.02	0.411 ± 0.01	0.903 ± 0.01	1.433 ± 0.01
*ρ* (tapped) (g/mL)	0.504 ± 0.01	0.490 ± 0.03	1.00 ± 0.01	1.58 ± 0.01
Flowability of the powder (g.s^−1^)	8.12 ± 0.25	7.23 ± 0.12	16.89 ± 0.23	19.72 ± 0.20
Hausner ratio (*HR*)	1.154 ± 0.02	1.192 ± 0.01	1.111 ± 0.01	1.105 ± 0.01
Diagnostic of powder according to (*HR*)	Sandy	Sandy	Sandy	Sandy
Carr’s ndex (*CI*)	0.133 ± 0.04	0.161 ± 0.02	0.100 ± 0.03	0.095 ± 0.02
Flowability according to (*CI*)	Excellent	Good	Excellent	Excellent
Angle of repose degrees (°)	31.62 ± 1.57	46.87 ± 1.00	41.03 ± 1.23	49.55 ± 1.71
Flowability according to angle of repose	Acceptable	Acceptable	Acceptable	Acceptable

**Table 2:** Spread ability of scrubs enriched with exhausted Obeidi grape seed residues at different concentrations (0, 0.5, 1, 1.5, and 2%), stored at 4, 25, and 50 °C. Mean values ± standard deviations were reported, *n* = 3. Same letters indicate not statistically different value (*p* > 0.05).

	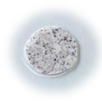	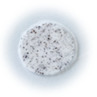	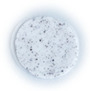	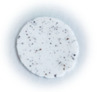	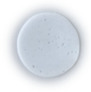
Scrubs	2%	1.5%	1%	0.5%	0%
Diameter at 4 °C (cm)	3.6 ± 0.1 a	3.7 ± 0.1 ab	3.7 ± 0.1 ab	4 ± 0.1 de	4.4 ± 0.1 g
Diameter at 25 °C (cm)	3.7 ± 0.1 ab	3.6 ± 0.1 a	4 ± 0.1 de	4.1 ± 0.1 ef	4.6 ± 0.1 h
Diameter at 50 °C (cm)	3.9 ± 0.1 cd	3.8 ± 0.1 bc	4.1 ± 0.1 ef	4.2 ± 0.1 f	4.7 ± 0.1 h

## Data Availability

Data is contained within the article.

## Sample Availability

Samples of the compounds are available from the authors under conditions.
